# Predicting nicotine dependence profiles among adolescent smokers: the roles of personal and social-environmental factors in a longitudinal framework

**DOI:** 10.1186/1471-2458-12-196

**Published:** 2012-03-16

**Authors:** Marloes Kleinjan, Frank Vitaro, Brigitte Wanner, Johannes Brug, Regina JJM Van den Eijnden, Rutger CME Engels

**Affiliations:** 1 Behavioural Science Institute, Radboud University Nijmegen, P.O. Box 9104, Nijmegen 6500 HE, The Netherlands; 2 Research Unit on Children's Psychosocial Maladjustment (G.R.I.P), Univerity of Montréal, 3050 Boulevard Edouard-Montpetit, Montréal, QC H3T 1J7, Canada; 3 EMGO Institute VU, University Medical Centre, Van der Boechorststraat 7, Amsterdam 1081 BT, The Netherlands; 4 Faculty of Social Sciences, Utrecht University, PO-Box 80.140, Utrecht 3508 TC, The Netherlands

**Keywords:** Adolescents, Smoking, Nicotine dependence, Personality, Parental/peer smoking

## Abstract

**Background:**

Although several studies have reported that symptoms of nicotine dependence can occur after limited exposure to smoking, the majority of research on nicotine dependence has focused on adult smokers. Insufficient knowledge exists regarding the epidemiology and aetiology of nicotine dependence among adolescent smokers. The objective of the present study is to identify the effects of theoretically driven social and individual predictors of nicotine dependence symptom profiles in a population-based sample of adolescent smokers.

**Method:**

A longitudinal study among 6,783 adolescents (12 to 14 years old at baseline) was conducted. In the first and second year of secondary education, personality traits and exposure to smoking in the social environment were assessed. Two and a half years later, adolescents' smoking status and nicotine dependence symptom profiles were assessed. A total of 796 adolescents were identified as smokers and included in the analyses.

**Results:**

At follow-up, four distinct dependence symptom profiles were identified: low cravings only, high cravings and withdrawal, high cravings and behavioural dependence, and overall highly dependent. Personality traits of neuroticism and extraversion did not independently predict nicotine dependence profiles, whereas exposure to smoking in the social environment posed a risk for the initial development of nicotine dependence symptoms. However, in combination with environmental exposure to smoking, extraversion and neuroticism increased the risk of developing more severe dependence symptom profiles.

**Conclusions:**

Nicotine dependence profiles are predicted by interactions between personal and environmental factors. These insights offer important directions for tailoring interventions to prevent the onset and escalation of nicotine dependence. Opportunities for intervention programs that target individuals with a high risk of developing more severe dependence symptom profiles are discussed.

## Background

Tobacco is a highly addictive substance. Clinical features of dependence can emerge even during the earliest phases of smoking initiation [1,2]. The occurrence of nicotine dependence symptoms among adolescent smokers forms an important barrier for smoking cessation [3-5]. Therefore, greater insights into the epidemiology and aetiology of nicotine dependence among adolescent smokers may have important implications for smoking prevention and cessation interventions among adolescents.

The most commonly used self-report measures of nicotine dependence, including the assessment proposed by the Diagnostic and Statistical Manual of Mental Disorders [6,7] and the Fagerström Tolerance Questionnaire (FTQ) [8], are not designed to measure the earlier stages of nicotine dependence; instead, they assume that a more established smoking pattern is required to yield the key features of nicotine dependence. Such characteristics make the measures less appropriate to assess dependence symptoms among adolescents as nicotine dependence symptoms may already be present among early smokers, but may not have reached a diagnosable level. Moreover, these measures generate a classification of low to high levels of dependence, implying that nicotine dependence varies only in severity and not necessarily in nature.

However, a recent study involving an adolescent smoking sample found that, when using a measure specifically developed to assess multiple features of nicotine dependence among adolescent smokers, distinct nicotine dependence symptom profiles could be distinguished [9]. By measuring symptoms indicative of behavioural dependence (when, where, and how much one smokes), craving, and withdrawal, it was shown that different patterns of symptoms were associated with increasing differences in severity of dependence. Results of this study defined four distinct, yet stable, nicotine dependence subtypes that could be characterized by quantitative as well as qualitative differences. For example, the presence of behavioural dependence appears to indicate more severe dependency than the presence of withdrawal symptoms alone [9]. In addition, a regular and more established smoking pattern did not seem to be a prerequisite for the occurrence of withdrawal symptoms; a substantial group of adolescents, despite displaying a considerable behavioural dependence and a high level of craving, did not seem to experience withdrawal symptoms. The symptom profiles were similar for both males and females, but differentially associated with previously identified correlates of nicotine dependence. Differential links of the four subtypes were found with regard to smoking uptake and cessation.

The identification of profiles in reference to nicotine dependence symptoms among adolescent smokers may provide some advantages over the use of counting symptoms to measure severity without taking the type of symptoms into account. First, the identification of different symptom profiles may enable a better understanding of the possible underlying genetic and psychosocial factors of nicotine dependence [10]. Twin studies suggest that genetically based differences in reaction to nicotine moderates the likelihood of taking up smoking [11,12]. Not everybody exposed to nicotine becomes dependent. Similarly, some are stimulated by nicotine, whereas others are calmed or even depressed by it [13]. Such differences in nicotine effects may be reflected in distinct symptom profiles. Second, classification of symptom profiles may enable the tailoring of intervention efforts to specifically target those symptoms most common among the different subtypes of adolescent smokers [14]. To increase insights into the aetiology of nicotine dependence, as well as design optimal ways of targeting nicotine dependence among adolescent smokers, it is essential to determine early predictors of nicotine dependence symptom profiles [9].

According to the Diathesis Stress model [15], the occurrence of problem behaviours or disorders is the result of the interaction between a vulnerable hereditary predisposition and precipitating events in the environment. Assuming that differences exist in the genetic basis of symptom profiles, different personality dimensions may predict nicotine dependence profiles. Personality has a strong genetic basis [16-18], and personality traits are reported to be associated with the initiation and maintenance of smoking in both adolescents and adults [19-21]. More specifically, several studies have found that smokers tend to be more neurotic and more extraverted than non-smokers. One possible explanation for this is that individuals scoring high on extraversion may smoke because they seek stimulation while those scoring high on neuroticism may smoke to reduce tension and anxiety [22-24]. With respect to adult smokers, researchers have suggested that, apart from smoking initiation and maintenance, personality traits such as neuroticism and extraversion may also be important in the development of dependence [25,26]. Thus, the first goal of the current study is to examine the effect of extraversion and neuroticism on the development of nicotine dependence symptom profiles in adolescent smokers.

As posited by Social Cognitive Theory [27], social environmental factors may also be important. With regard to smoking behaviour in adolescents, exposure to smoking by significant others is related to the development of nicotine dependence symptoms. Children had a higher risk of becoming nicotine dependent from adolescence to early adulthood when their mother had ever smoked, been a daily smoker, or was dependent on nicotine [28,29]. Having smoking peers was also associated with higher levels of nicotine dependence in adolescents [28,30,31]. Meanwhile, the Diathesis Stress Model indicates that the smoking of parents and peers may also exacerbate or precipitate the links between personal dispositions and nicotine dependence according to a person-environment interactional perspective. Hence, the second goal of the proposed study is to examine the additive and interactive effect of having a smoking mother or having smoking friends in addition to the personality traits of extraversion and neuroticism.

To pursue these study goals, the current study examines individual and environmental factors as possible predictors of nicotine dependence symptom profiles among adolescent smokers. A clearer understanding of these factors can provide an important foundation for and contribution to developing effective tailored intervention methods for targeting smoking among adolescents.

## Methods

### Participants

The data of the present study pertain to a population-based cohort study initiated in January 2003 as part of the International Study of Asthma and Allergies in Childhood (ISAAC) phase III. Schools in four regions of the Netherlands were randomly selected from the telephone book and approached about participating. The main reason given by those schools declining to participate in this study was participation in other studies. The present study pertains to two measurement waves, including a total of 25 schools. Data for the first wave (T1) were collected from January 2003 to May 2003, in the first and second year of secondary education. The completion rate was 89.7% among the total sample, resulting in 6,783 respondents who were 12 to 14 years old (M = 12.88, SD = 0.76). The second measurement wave (T2) described in this study took place approximately 2.5 years later in November 2005. A total of 4,270 respondents of the original 6,783 respondents participated in this wave (response rate, 63%). Sickness, truancy, relocation to another school, the repeating of a class, and graduation from school (the latter accounting for 52% of the total loss to follow-up) were noted by teachers as the primary causes for non-response. With regard to the last cause of attrition, it should be noted that at the time of T2, respondents who were in their fourth year of preparatory vocational training and junior general secondary training, or their fifth year of senior general secondary education, had already graduated. When possible, these respondents were contacted at home and asked to fill out the questionnaire. The medical ethical committee (CMO Arnhem-Nijmegen) approved this study.

At T1, a total of 219 of the 4,270 respondents (5.2%) indicated smoking at least once a month. At T2, a total of 796 (18.6%) indicated that they had smoked at least once in the past month. Of the 219 smokers at T1, 169 were also classified as smokers at T2 (77.2%). Because the different aspects of nicotine dependence were only assessed for respondents who indicated that they had smoked during the previous month (non-smokers were instructed to skip this section), the 796 respondents who indicated that they had smoked at T2 were included in the main analyses. Of the 796 smoking respondents included in the present study, just over half (56.8%) were female. In addition, among all 796 smoking respondents, 33.4% received preparatory vocational training, 21.3% junior general secondary training, 28.6% senior general secondary education, 15.7% university preparatory training, and 0.9% some other form of education.

### Procedure

Respondents completed written, self-administered questionnaires in the presence of their teacher during school hours. Students were informed that the data would be processed anonymously as respondent-specific codes would be used to link the data from one point in time to the next. To ensure confidentiality, each student received an unmarked envelope in which to return the completed questionnaires. In addition, respondents were informed that participation was voluntary.

### Attrition Analyses

Of the 6,783 respondents at T1, 4,270 were included again at T2. The respondents lost at follow-up were compared with the remaining respondents in regards to gender, age, education, and smoking status using multivariable logistic regression analyses. Logistic regression analysis with loss to follow-up (No/Yes) as a dependent variable demonstrated that respondents lost at follow-up were significantly more likely to be boys, have general secondary training, and be smokers (Nagelkerke R^2 ^= 0.06).

### Measures

#### Personality dimensions of extraversion and neuroticism

The personality dimensions of extraversion and neuroticism assessed at T1 were each measured using six items. The items assessing extraversion and neuroticism were part of the Quick Big Five, a well-validated instrument that aims to assess the factors of the Five Factor Model of personality [32]. Respondents were asked to indicate on a 7-point scale to what degree they possessed certain traits distinctive of either extraversion or neuroticism. Extraversion was measured by items such as being quiet, shy, or withdrawn (Cronbach's alpha = 0.68). The items were re-coded so that a higher score on this scale represented a higher level of extraversion. Neuroticism was measured by items such as being fearful, nervous, or sensitive (Cronbach's alpha = 0.71).

#### Smoking mother

The smoking status of the respondent's mother at T1 was assessed using a yes/no question: "Does your mother smoke?". Adolescents' proxy reports on parental smoking were found to be reliable indicators of parents' lifetime and current smoking status [33].^a^

#### Smoking friends

Smoking of friends at T1 was assessed by asking adolescents to estimate the proportion of smoking friends on a 5-point scale, ranging from 1 ('none of my friends smoke') to 5 ('all of my friends smoke') [34]. At T1, 54.5% of the participants indicated having smoking friends.

#### Nicotine dependence

The different aspects of nicotine dependence were assessed at T2 for smokers only, using a newly developed multidimensional scale based on both the modified Fagerström Tolerance Questionnaire (mFTQ) and the Hooked on Nicotine Checklist (HONC) [35,36]. The 11-item scale was validated in a previous study [37]. The HONC was specifically developed for use among adolescent smokers whose dependence is still developing [38]. The mFTQ focuses on measuring behavioural aspects and strength of physical dependence; it was not designed to measure the earlier stages of nicotine dependence [39,40]. The combination of the mFTQ and the HONC included items thought to be indicative of early symptoms as well as items presumably indicative of symptoms that occur when dependence is more manifest; taken together, these items enable the measurement of a wider range of nicotine dependence. Cronbach's alpha for this scale was .89 in the current study.

#### Nicotine dependence profiles

A previous study by Kleinjan and colleagues [9] demonstrated that, based on the combined items, four distinct profiles of nicotine dependence could be identified among the adolescent smoking population. One profile was composed of adolescents who displayed low cravings only. The second profile was composed of adolescents who displayed high cravings and withdrawal. The third profile was composed of adolescents displaying high cravings and behavioural dependence. The fourth profile displayed high scores on cravings, behavioural dependence, and withdrawal. One year later, when nicotine dependence symptoms were assessed again, the coefficients of the loadings on the latent variables were consistent over time. Furthermore, latent transition analyses showed that very few adolescents transferred from either the high cravings and behavioural dependence profile and the overall high dependence profile to the other two (i.e., low cravings only or high craving and withdrawal). Adolescents had a relatively high chance of transferring from the high cravings and withdrawal profile to the high cravings and behavioural dependence profile or the overall high dependence profile. As for the low cravings only category, a large proportion transferred to the high cravings and withdrawal profile. Furthermore, results showed that adolescent smokers in the low cravings only category had the highest likelihood of being a non-smoker at the follow-up measure, followed by the high cravings and withdrawal category, high cravings and behavioural dependence category, and the overall high dependence category. These results indicate that the four qualitatively distinct profiles quantitatively differ with regard to severity of dependence, with the low cravings only profile being the least severe dependence profile, followed by the high cravings and withdrawal profile, the high cravings and behavioural dependence profile, and the overall high dependence profile, respectively [9]. Descriptions of the 11 nicotine dependence items are included in Table 1.

**Table 1:** Descriptions and characteristics of dependent and independent variables

Measurement	Response categories	T1	T2
Smoking frequency	1. Daily	1. 9.4%	1. 58.4%
	2. Weekly	2. 3.7%	2. 13.6%
	3. Monthly	3. 2.0%	3. 9.1%
	4. Less than once a month	4. 1.8%	4. 3.4%
	5. Only experimented	5. 6.5%	5. 15.4%
	6. Never, not even one puff	6. 76.5%	n.a.
Smoking quantity per week		M = 3.63	M = 37.15
		SD = 15.10	SD = 45.76
Length of time smoking (years)			M = 3.52
			SD = 2.37
With whom do you live?	1. Both parents	1. 84.5%	
	2. Mother	2. 10.6%	
	3. Father	3. 1.6%	
	4. Other	4. 3.2%	
Extraversion	1 'not at all' to 7. 'very much'	M = 4.85	
				SD = .92	
Neuroticism	1 'not at all' to 7. 'very much'	M = 3.72	
				SD = .98	
Maternal smoking	1. Smoking mother	1. 40.5%	
	2. No smoking mother	2. 59.5%	
Paternal smoking	1. Smoking father	1. 42.7%	
	2. No smoking father	2. 57.3%	
Number of friends smoking	1. None	1. 45.4%	
	2. Less than half 3. Half	2. 35.1% 3. 7.8%	
	4. More than half	4. 9.8%	
	5. All	5. 1.5%	
**Behavioural aspects of nicotine dependence**			
B1: How soon after you wake up do you smoke your first cigarette	1. After 60 minutes		1. 61.1%
	2. Within 31-60 minutes		2. 15.8%
	3. Within 6-30 minutes		3. 15.7%
	4. Within 5 minutes		4. 7.4%
B2: How many cigarettes do you smoke in a day?	1. Fewer than 1 a day		1. 35.3%
	2. About 1-5 a day		2. 21.5%
	3. About 6-10 a day		3. 22.2%
	4. About 11-20 a day		4. 17.3%
	5. About 21-30 a day		5. 2.3%
	6. More than 30 a day		6. 1.4%
B3: Which cigarette would you hate to give up?	1. Any other cigarette		1. 74.0%
	2. First in the morning		2. 26.0%
B4. Do you smoke if you are so ill that you are in bed most of the day?	1. No		1. 84.4%
	2. Yes		2. 15.6%
**Craving** C1: Have you ever felt like you were addicted to tobacco?	1. Never		1. 33.5%
	2. Seldom		2. 17.1%
	3. Sometimes		3. 30.8%
	4. Often		4. 18.5%
C2: Do you ever have strong cravings to smoke?	1. Never		1. 9.4%
	2. Seldom		2. 19.5%
	3. Sometimes		3. 45.6%
	4. Often		4. 25.4%
C3: Have you ever felt like you really needed a cigarette?	1. Never		1. 24.5%
	2. Seldom		2. 20.0%
	3. Sometimes		3. 37.5%
	4. Often		4. 17.6%
C/W4: Do you smoke because it is really hard to quit?	1. No, not at all		1. 60.7%
	2. A little		2. 20.6%
	3. Quite		3. 14.5%
	4. Yes, very		4. 3.7%
**Withdrawal**			
W1: Trouble concentrating	1. Never		1. 62.9%
	2. Seldom		2. 17.8%
	3. Sometimes		3. 13.3%
	4. Often		4. 5.0%
W2: Feeling irritable or angry	1. Never		1. 58.8%
	2. Seldom		2. 16.3%
	3. Sometimes		3. 17.8%
	4. Often		4. 6.2%
W3: Feeling nervous, restless or anxious	1. Never		1. 69.6%
	2. Seldom		2. 17.6%
	3. Sometimes		3. 9.1%
	4. Often		4. 2.8%

C/W4 = Do you smoke now because it is really hard to quit? The answer to this item can be regarded as being a result of both craving and withdrawal symptoms

**Table 2:** Pearson and Spearman correlations among control variables, personality traits, mother's and friends' smoking, and nicotine dependence profiles (N = 796)

	1	2	3	4	5	6	7	8	9
1. Sex	_								
2. Education	**-.05**	_							
3. Age of initiation	**-.01**	.19***	_						
4. Smoking frequency	**.05**	-.15***	-.25***	_					
5. Smoking quantity	**.05**	-.17***	-.23***	.58***	_				
6. Extraversion	**.04**	.13***	*-.00*	**.06**	**.01**	_			
7. Neuroticism	**.07**	**-.04**	*-.02*	**-.01**	**-.01**	-.51***	_		
8. Smoking mother	**.05**	-.28***	-.12**	.18***	.14***	**.02**	**.01**	_	
9. Smoking friends	.10**	-.17***	-.22***	.55***	.53***	**.03**	**-.05**	.14***	_
10. Nicotine dependence profiles	**-.00**	-.30***	-.36***	.25***	.26***	**-.02**	.06**	.17***	.24***

Pearson correlations are in italics, Spearson correlations are in boldface

* *p *< .05, **p < .01, *** *p *< .001

### Statistical Analyses

The analyses proceeded in two steps. The first step was based on the previous study by Kleinjan and colleagues [9], in which latent class analysis (LCA) was applied to examine whether empirically derived classes of nicotine dependence could be identified within a population sample of adolescent smokers. The present study sought to replicate these results within a larger and older sample of adolescent smokers from the same cohort using the software package MPLUS 4.1 [41]. Table 1 provides a detailed description of the items used at T2 to generate the latent classes. All items with answer categories not scaled to range from 1 to 4 were rescaled to range between 1 and 4 for the analyses, thereby ensuring that each item contributes an equal amount of weight to the scale. For a detailed description of the LCA procedure, we refer to Kleinjan and colleagues [9].

Second, chunk-wise multinomial logistic regression analyses using SPSS were performed to predict adolescents' membership in one of the nicotine dependence classes at T2. The alternative to multinomial logistic regression, ordinal logistic regression, is not ideal given that qualitative differences between classes are expected, and contrary to ordinal logistic regression, multinomial regression allows the odds for class comparisons to vary. To account for uncertainty of membership in the subclasses, we used the posterior probabilities of being members in the respective subclass as weights in the multinomial regression analyses [42,43]. In the first step, sex, education level, age of smoking initiation, smoking frequency, and smoking quantity as measured at T1 were included as control variables. All these variables were found to be associated with nicotine dependence in previous studies [28,44-46]. In the second step, we included the personality dimensions of extraversion and neuroticism as predictors. In the third step, the smoking of mother and smoking of friends were added as predictors. Finally, in the fourth step, the interaction terms of the personality dimensions with smoking of mother and friends were added.

For each step, comparisons were made regarding the covariates' scores in the one category as compared to all other categories. However, when depicting the results, we will use the low cravings only category as the comparison group for the high cravings and withdrawal category, the high cravings and withdrawal category as the comparison for the high cravings and behavioural dependence category, and the high cravings and behavioural dependence category as the comparison for overall high dependence category. We thus do not depict all comparisons that were made within the multinomial regression analyses. We chose to depict this limited number of statistical comparisons for reasons of interpretability and because the empirical patterns of results in our previous study [9] as well as in the present study support the idea that the low cravings only profile (class 1) is the least severe dependence profile, followed by the high cravings and withdrawal profile (class 2), the high cravings and behavioural dependence profile (class 3), and the overall high dependence profile (class 4), respectively.

To avoid chance capitalization because of the multiple comparisons, Bonferroni correction was applied; the alpha was considered significant when it fell below 0.02.

In addition, one might assume that respondents attending the same school are likely to produce common sources of variance, which could violate the accuracy of the effects. To obtain an indication of this design effect we conducted binary logistic regressions comparing two classes (class 1 vs class 2, class 2 vs. class 3 and class 3 vs. class 4, respectively) on the full model with all predictor variables as a (1) simple model and as (2) a multilevel model with schools as clusters in Mplus. Subsequently, we compared the variance of the estimates of the regression coefficients under model (1) and (2). These analyses show that results of the simple models and the multilevel models, respectively, are highly comparable. Based on these findings, we assume that the use of multilevel analyses would not provide a substantial improvement in statistical analyses. Spearman correlations between the ordinal variable nicotine dependence classes (1 = low cravings only; 2 = high cravings and withdrawal; 3 = high cravings and behavioural dependence; 4 = overall high dependence) and all model variables will be provided to facilitate a more detailed insight into the univariate associations.

## Results

### Descriptive statistics

Information about family composition (i.e., what proportion of participants live full time with their mother), smoking quantity and frequency, average length of time smoking, and the proportion of adolescents endorsing each dependence symptom is provided in Table 1.

### Identification of nicotine dependence subclasses

**Figure 1:**
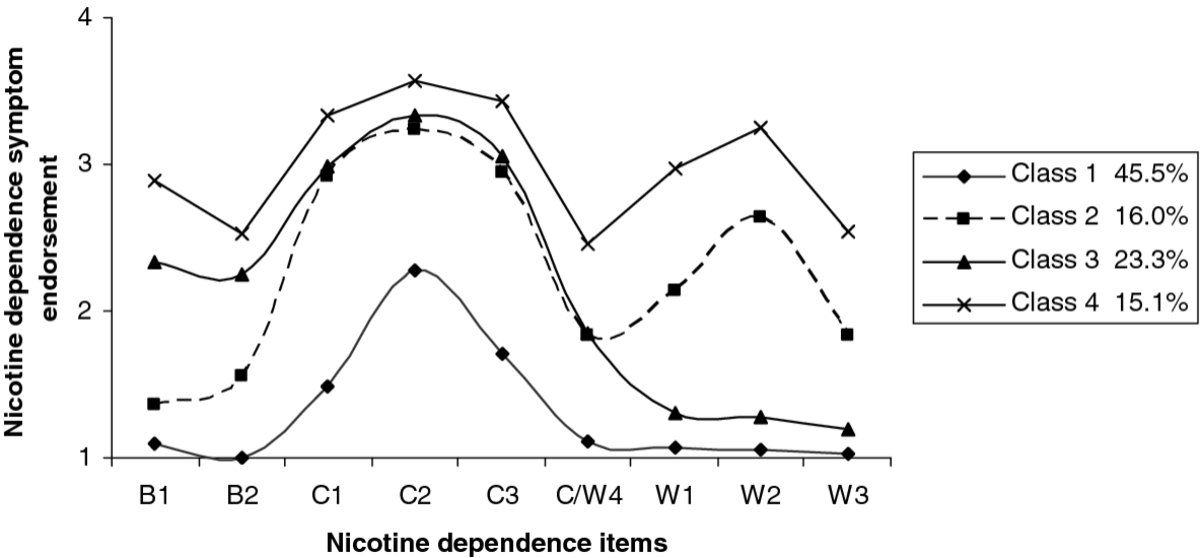
**Latent class profiles depicting the endorsement of the nicotine dependence items at T2 for the four derived subtypes**.

Results from the LCA showed that the nicotine dependence symptom profiles identified by Kleinjan and colleagues [9] were replicated. The latent class membership statistics for the two nominal items "Which cigarette would you hate to give up?" and "Do you smoke if you are so ill that you are in bed most of the day?"--are described in Table 3 and the latent class profile for the continuous items is depicted in Figure 1, which shows that the profiles representing the low cravings only category and the overall high dependence category mainly reflect differences in degree of dependence. However, the profiles representing the high cravings and withdrawal category and the high cravings and behavioural dependence category show differences in the likelihood of endorsing certain symptoms. The overall high dependence and high cravings and behavioural dependence categories report similar symptoms indicative of behavioural dependence, but these two groups seem to differ in endorsement of withdrawal symptoms, suggesting that the profiles do not merely differ along a severity dimension. In the present study, the low cravings only category was approximately 45.5% of smokers. The high cravings and withdrawal category accounted for approximately 16.0%. The high cravings and behavioural dependence category consisted of 23.3% of all smokers, whereas the overall high dependence category consisted of 15.1%.

**Table 3:** Item response probabilities per identified latent class for the two nominal items assessing behavioural aspects of nicotine dependence

Item response probabilities^a^		
**Latent classes identified**	**Item B3**	**Item B4**

Low cravings only	0.05	0.01
High cravings and withdrawal	0.21	0.05
High cravings and behavioural dependence	0.46	0.28
Overall high dependence	0.64	0.52

^a ^Probability of responding to the answer category indicative of dependence

*Item B3 = *Which cigarette would you hate to give up?; Item B4 = Do you smoke if you are so ill that you are in bed most of the day?

### Correlations between predictor variables and nicotine dependence profiles

Spearman correlations indicated that the nicotine dependence profiles were negatively associated with education level and age of smoking initiation, whereas positive associations were found between the subclasses and smoking frequency and quantity (see Table 2). No associations were found between the personality traits extraversion and neuroticism and the nicotine dependence profiles, whereas the smoking of both the mother and friends was positively associated with the profiles.

### Multinomial logistic regression analyses

Table 4 presents the outcomes of the chunk-wise multinomial logistic regression analyses. No main effects were found for the personality dimensions of extraversion and neuroticism on the endorsement of the dependence profiles. The inclusion of the main effects of environmental smoking indicated that participants were more likely to be classified in the high cravings and withdrawal category than in the low cravings only category if they had a smoking mother (*Odds Ratio = *1.95, *p *< 0.02). In addition, participants were more likely to be classified in the high cravings and behavioural dependence category as opposed to the high cravings and withdrawal category if they had a smoking mother (*Odds Ratio *= 2.25, *p *< 0.01). Participants who reported having a greater share of smoking friends were more likely to be classified in the high cravings and withdrawal category as opposed to the low cravings only category (*Odds Ratio *= 1.47, *p *< 0.01). Furthermore, although no main effect of the personality dimensions of extraversion and neuroticism existed, the inclusion of the interaction terms in the fourth step of the personality dimensions × smoking of mother or share of smoking friends indicated a significant interaction effect of the share of smoking friends regarding the link between extraversion and the likelihood of being classified in the high cravings and withdrawal category as opposed to the low cravings only category (*Odds Ratio = *1.48; *p *< 0.01). In addition, a significant interaction effect was found for having a smoking mother regarding the link between extraversion and the likelihood of being classified in the overall high dependence category as opposed to the high cravings and behavioural dependence category (*Odds Ratio = *1.79; *p *< 0.02). Table 4 depicts only the significant interaction effects.

**Table 4:** Odds Ratios (OR) and 95% confidence intervals (CI) for a multinomial regression comparison of the four nicotine dependence subclasses with personality traits, maternal and peer smoking, and interaction effects as predictors (N = 796)

	Nagelkerke R^2 ^- Change	Reference Class		OR (95% CI)
Step 1	0.159			
Sex		Class 1	Class 2	1.04 (.66-1.63)
		Class 2	Class 3	0.70 (.43-1.15)
		Class 3	Class 4	1.17 (.71-1.93)
Education		Class 1	Class 2	0.74*** (.65-.83)
		Class 2	Class 3	1.01 (.89-1.05)
		Class 3	Class 4	1.04 (.90-1.19)
Age of initiation		Class 1	Class 2	1.02 (.94-1.11)
		Class 2	Class 3	0.96 (.73-1.26)
		Class 3	Class 4	1.04 (.81-1.33)
Smoking frequency		Class 1	Class 2	.99 (.75-1.33)
		Class 2	Class 3	.96 (.73-1.26)
		Class 3	Class 4	1.04 (.81-1.33)
Smoking quantity		Class 1	Class 2	1.93 (.61-6.10)
		Class 2	Class 3	1.49 (.56-4.01)
		Class 3	Class 4	1.19 (.51-2.77)
Step 2	0.010			
Extraversion		Class 1	Class 2	0.87 (.67-1.13)
		Class 2	Class 3	1.07 (.81-1.43)
		Class 3	Class 4	1.18 (.87-1.58)
Neuroticism		Class 1	Class 2	1.16 (.90-1.51)
		Class 2	Class 3	0.88 (.66-1.17)
		Class 3	Class 4	1.13 (.85-1.51)
Step 3	0.035			
Smoking mother		Class 1	Class 2	1.95* (1.17-3.26)
		Class 2	Class 3	2.25** (1.30-3.87)
		Class 3	Class 4	1.34 (.79-2.25)
Smoking friends		Class 1	Class 2	1.47** (1.12-1.94)
		Class 2	Class 3	.98 (.74-1.31)
		Class 3	Class 4	.94 (.70-1.25)
Step 4a	0.010			
Interaction Extraversion × Smoking friends		Class 1	Class 2	1.48** (1.13-1.96)
Step 4b	0.011			
Interaction Extraversion × Smoking mother		Class 3	Class 4	1.79* (1.08-2.97)

* p < 0.02; ** p < 0.01; ***p < 0.001.

Sex is coded such that 0 indicates boys and 1 indicates girls. Only significant interactions are included in the table. Class 1 = 'low cravings only'; Class 2 = 'high cravings and withdrawal; Class 3 = 'high cravings and behavioural dependence'; Class 4 = 'overall high dependence'

To interpret the interaction effects, we repeated the analyses stratified by having a smoking friend and by having a smoking mother [47]. Extraversion predicted a higher likelihood of being classified in the high cravings and withdrawal category than in the low cravings only category among adolescents with smoking friends (*Odds Ratio = *1.40; *p *< .02). The interpretation of the two interaction effects revealed that extraversion predicted the likelihood to be classified in the highest dependence category as opposed to the high cravings and behavioral dependence class, only among adolescents with a smoking mother (*Odds Ratio = *1.42; *p <*0.02).

## Discussion

Among adolescent smokers, individual differences emerged in the susceptibility to nicotine dependence symptoms. In agreement with an earlier study [9], the present study showed that four symptom profiles can be distinguished according to items indicative of behavioural dependence, cravings, and withdrawal symptoms. Furthermore, this study demonstrated that personal as well as social-environmental factors predict these distinct profiles.

Although the personality traits of neuroticism and extraversion were found to be predictive of smoking initiation and regular smoking in adolescents [20,21], they do not seem to form independent risk factors for the development of dependence symptom profiles. On the other hand, in line with the Social Cognitive Theory [27], exposure to parents' or friends' smoking serves as an important risk factor for the development of future dependence symptoms [27]. These findings corroborate previous findings suggesting that the social context is more strongly related to adolescent smoking and the initial development of nicotine dependence symptoms compared to personality traits [21,48]. However, in line with the Diathesis Stress Model [15], personality traits did interact with the exposure to smoking behaviour of significant others in explaining differences in dependence symptom profiles. When combined with environmental exposure to smoking, extraversion seems to increase the risk of developing withdrawal symptoms in addition to craving when adolescents have smoking friends and a higher score on all three features of dependence when adolescents have a smoking mother. The findings of the present study suggest that being extraverted in combination with exposure to smokers in the environment may increase the risk for developing dependence symptoms. Thus far, person-environment interactions have predominantly been investigated in relation to adolescent smoking initiation [21,48] rather than smoking persistence and the development of nicotine dependence symptoms.

### Implications

The identification of precursors of dependence symptom profiles may prove particularly helpful to differentially prevent or remedy nicotine dependence by targeting specific precursors of the various subtypes. Being extraverted in combination with the presence of smoking friends seems to coincide with the initial occurrence of withdrawal symptoms in addition to craving. Being around smoking friends may enhance exposure to smoking cues that trigger craving and withdrawal symptoms [49]. The reporting of behavioural dependence symptoms in addition to craving and withdrawal seems to be linked to being extraverted in combination with having a smoking mother. Having a smoking mother and or smoking friends may create more withdrawal and behavioural dependence-enhancing circumstances, such as ample availability or offering of cigarettes and occasions to smoke. Because of their outgoing nature, extraverted adolescents may be more vulnerable to social influence or more susceptible to adopting peer behaviours (e.g., showing similar behaviours and engaging in similar activities are rewarding). Making extraverted smokers aware of their increased vulnerability to social influences with regard to smoking and the risk of developing dependence symptoms may encourage them to avoid (or be particularly cautious about) risky social settings. Hence, interventions could aim at stimulating ways of instrumental support that the direct environment can provide to discourage smoking, such as not smoking in the presence of someone who is attempting to quit. Previous research has indicated that instrumental support leads to the lower likelihood of substance use in adolescents [50]. In addition, actions to prohibit smoking in social settings (such as school grounds, bars, restaurants and other public places) may be effective in decreasing nicotine dependence and enhancing quitting among adolescents.

To prevent classification in high dependence symptom profiles, it is recommended that intervention programs be designed to more specifically target individuals with a high risk of developing dependence symptom profiles characterized by multiple features of dependence and a higher endorsement of these features (e.g., individuals exposed to environmental smoking), particularly if they have an extraverted personality. Prevention efforts based on personality risk profiles have already been applied with some success to target adolescent drinking behaviour and binge drinking [51,52]. One example of such a targeted approach is the Preventure program. Tailoring the intensity and type of smoking treatment may be more effective for these high risk individuals compared to the more widely implemented general approaches. Given the generally low levels of funding available for public health programs, the Internet might provide an especially suitable platform to facilitate this. Brief web-based interventions could be designed that provide tailored feedback program for adolescents based on a short examination of the extent to which they experience symptoms of nicotine dependence and adhere to certain personality characteristics. The Internet is easily accessible and particularly appealing to young people; they can complete the test in the privacy of their homes at a time convenient for them. In addition, the costs of web-based interventions are relatively low.

### Limitations

The present study has several limitations. First, the data are based on adolescents' self-reports of their own smoking and that of their parents and friends; thus, under- or over-reporting may have occurred [53,54]. However, self-reported smoking behaviour has been found to be reliable and valid compared with more objective methods, such as biochemical validation [55-57].

Second, attrition analysis indicated a possible under-representation of lower educated adolescent male smokers. Since a lower educational level has been associated with higher levels of nicotine dependence [28], caution is warranted when interpreting and generalizing the findings of the present study to the general adolescent smoking population.

Third, although the present study explored individual characteristics and peer and family factors in the emergence of nicotine dependence symptoms, these predictors are by no means exhaustive. Personality traits other than neuroticism and extraversion were not taken into account, nor did we include a full spectrum of environmental factors, such as siblings' smoking or smoking in the media. However, the present study tested clearly specified and theoretically driven predictors found to have explanatory value with regard to smoking behaviour among adolescents, even when controlled for intensity and frequency of cigarette use, which were found to be strong predictors of the development of smoking trajectories and nicotine dependence in previous studies [45,46,58].

Fourth, smoking frequency is used as an indicator of nicotine dependence (categorical variable: cigarettes per day) as well as a control variable (continuous variable: cigarettes per week). Because smoking frequency--just like smoking quantity and length of time smoking--is likely to be confounded with the dependence profiles and the association of personality characteristics and social environment with the profiles, we chose to include smoking frequency as well in the multinomial regression analyses in order to examine the unique associations for the personality and environmental variables. Analyses were run both with and without smoking frequency as a control variable; results were similar in both approaches.

Finally, some caution is warranted when describing the effect of maternal smoking as solely an environmental risk factor. Maternal smoking may influence nicotine dependence through a modelling effect, but genetic transmission or perinatal factors triggered by maternal smoking may also be partly responsible for the development of specific symptoms.

### Directions for future research

Increasing evidence suggests that nicotine dependence symptoms are substantially heritable [30,59,60]. The identification of nicotine dependence phenotypes may facilitate future research on genetic causes of behaviour, such as by testing the different phenotypes for an association with a particular genetic factor. As important genetic factors of nicotine dependence are identified, we can examine how they interact with environmental influences in determining addictive behaviour. The identification of specific genetic and environmental mechanisms that underlie the emergence of dependence symptoms among novice adolescent smokers will likely lead to a more refined understanding of the aetiology of nicotine dependence.

## Conclusions

Despite the potential limitations, our findings help explain the emergence of variability in adolescent nicotine dependence profiles and may provide indications as to why some adolescents develop a full dependence syndrome while others do not. The present study provides insights into individual and environmental factors and mechanisms underlying the development of nicotine dependence symptom profiles in adolescents. These insights may prove important in efforts to tailor optimal interventions to prevent the onset and escalation of nicotine dependence in adolescents.

## Endnotes

^a^We also conducted analyses, including smoking behaviour of the father. However, smoking of the father at baseline was not significantly related to the nicotine dependence symptom profiles at follow-up. In addition, combining smoking behaviour of the father and mother into one variable also showed no significant links to the outcome variable. To ease the interpretability of the results, we decided not to include smoking of the father in the final model.

## Competing interests

The authors declare that they have no competing interests.

## Authors' contributions

RE and RvdE designed the longitudinal study and wrote the protocol. MK, FV, and BW developed the specific research questions and hypothesis discussed in the manuscript. MK managed the literature searches and summaries of previous related work. MK and BW undertook the statistical analysis. All authors were involved in the critical revision of the first draft. All authors contributed to and have approved the final manuscript.

## Pre-publication history

The pre-publication history for this paper can be accessed here:

http://www.biomedcentral.com/1471-2458/12/196/prepub

## References

[B1] DiFranza JR Rigotti NA McNeill AD Ockene JK Savageau JA St Cyr D Coleman M Initial symptoms of nicotine dependence in adolescents Tob Control 2000 9 313 319 10.1136/tc.9.3.313 10982576 PMC1748379

[B2] O'Loughlin JO DiFranza J Tyndale RF Meshefedjian G McMillan-Davey E Clarke P Hanley J Paradis G Nicotine-dependence symptoms are associated with smoking frequency in adolescents Am J Prev Med 2003 25 219 225 10.1016/S0749-3797(03)00198-3 14507528

[B3] Prokhorov AV Suchanek Hudmon K De Moor CA Kelder SH Conroy JL Ordway N Nicotine dependence withdrawal symptoms and adolescents' readiness to quit smoking Nicotine Tob Res 2001 3 151 155 11403729 10.1080/14622200110043068

[B4] Kleinjan M Brug J Van den Eijnden RJJM Vermulst AA Van Zundert RMP Engels RCME Associations between the Transtheoretical processes of change nicotine dependence and adolescent smoking cessation Addiction 2008 103 331 338 10.1111/j.1360-0443.2007.02068.x 18199313

[B5] Kleinjan M Van den Eijnden RJJM Van Leeuwe J Brug J Van de Ven MOM Engels RCME Adolescents' movement towards cessation of smoking: Role and relative value of the processes of change and nicotine dependence Psychol Health 2008 23 729 743 10.1080/08870440701757344 25160813

[B6] American Psychiatric Association Diagnostic and statistical manual of mental disorders (revised 3rd ed) 1987 Washington DC: Author

[B7] American Psychiatric Association Diagnostic and statistical manual of mental disorders (revised 4th ed) 1994 Washington DC: Author

[B8] Fagerström KO Schneider NG Measuring nicotine dependence: A review of the Fagerström Tolerance Questionnaire J Behav Med 1989 12 159 182 10.1007/BF00846549 2668531

[B9] Kleinjan M Wanner B Vitaro F Van den Eijnden RJJM Brug J Engels RCME Nicotine dependence subtypes among adolescent smokers: Examining the occurrence development and validity of distinct symptom profiles Psychol Addict Behav 2010 24 61 74 20307113 10.1037/a0018543

[B10] Storr CL Reboussin BA Anthony JC The Fagerstrom test for nicotine dependence: A comparison of standard scoring and latent class analysis approaches Drug Alcohol Depend 2005 80 241 250 10.1016/j.drugalcdep.2004.04.021 15908142

[B11] Epstein LH Grunberg NE Lichtenstein E Evans RI Smoking research: Basic research intervention prevention and new trends Health Psychol 1989 8 705 721 2700344

[B12] Vink JM Beem AL Posthuma D Neale MC Willemsen G Kendler KS Slagboom PE Boomsma DI Linkage analysis of smoking initiation and quantity in Dutch sibling pairs Pharmacogenetics 2004 4 274 282 10.1038/sj.tpj.6500255 15170444

[B13] Pomerleau OF Pomerleau CS Neuroregulators and the reinforcement of smoking: towards a biobehavioral explanation Neurosci Biobehav Rev 1984 8 503 513 10.1016/0149-7634(84)90007-1 6151160

[B14] Xian H Scherrer JF Eisen SA Lyons MJ Tsuang M True WR Bucholz KK Nicotine dependence subtypes: Association with smoking history diagnostic criteria and psychiatric disorders in 5440 regular smokers from the Vietnam Era Twin Registry Addict Behav 2007 32 137 147 10.1016/j.addbeh.2006.03.031 16647217

[B15] Zubin J Spring B Vulnerability: a new view of schizophrenia J Abnorm Psychol 1977 86 103 126 858828 10.1037//0021-843x.86.2.103

[B16] Bouchard TJ Genetic influence on human psychological traits: A survey Curr Dir Psychol Sci 2004 13 148 151 10.1111/j.0963-7214.2004.00295.x

[B17] Bouchard TJ Jr Loehlin JC Genes evolution and personality Behav Genet 2001 31 243 273 10.1023/A:1012294324713 11699599

[B18] Turkheimer E Three laws of behavior genetics and what they mean Curr Dir Psychol Sci 2000 13 160 164

[B19] Cherry N Kiernan K Personality scores and smoking behavior Br J Prev Soc Med 1976 30 123 131 953376 10.1136/jech.30.2.123 PMC478950

[B20] Harakeh Z Scholte RHJ De Vries H Engels RCME Association between personality and adolescent smoking Addict Behav 2006 31 232 245 10.1016/j.addbeh.2005.05.003 15953689

[B21] Otten R Engels RCME Van den Eijnden RJJM Smoking behaviour in asthmatic and non-asthmatic adolescents: the role of smoking models and personality Subst Use Misuse 2008 43 341 360 10.1080/10826080701202833 18365936

[B22] Eysenck HJ The causes and effects of smoking 1980 Beverly Hills CA: Sage

[B23] Pritchard WS Electroencepholographic effects of cigarette smoking Psychopharmacology 1991 104 485 490 10.1007/BF02245654 1780419

[B24] Fowler JS Volkow ND Wang GJ Pappas N Logan J MacGregor R Alexoff D Shea C Schlyer D Wolf AP Warner D Zezulkova I Cilento R Brain monoamine oxidase: An inhibition in cigarette smokers Proc Natl Acad Sci USA 1996 93 14065 14069 10.1073/pnas.93.24.14065 8943061 PMC19495

[B25] Breslau N Johnson EO Hiripi E Kessler R Nicotine dependence in the United States: prevalence trends and smoking persistence Arch Gen Psychiatry 2001 58 810 816 10.1001/archpsyc.58.9.810 11545662

[B26] McChargue DE Cohen LM Cook JW The influence of personality and affect on nicotine dependence among male college students Nicotine Tob Res 2004 6 287 294 10.1080/14622200410001676323 15203802

[B27] Bandura A Social foundations of thought and action: A social cognitive theory 1986 Englewood Cliffs NJ: Prentice Hall

[B28] Hu MC Davies M Kandel DB Epidemiology and correlates of daily smoking and nicotine dependence among young adults in the United States Am J Public Health 2006 96 299 308 10.2105/AJPH.2004.057232 16380569 PMC1470478

[B29] Lieb R Schreier A Pfister H Wittchen HU Maternal smoking and smoking in adolescents: a prospective community study of adolescents and their mothers Eur Addict Res 2003 9 120 130 10.1159/000070980 12837990

[B30] Audrain-McGovern J Koudsi NA Rodriguez D Wileyto EP Shields PG Tyndale RF The Role of CYP2A6 in the Emergence of Nicotine Dependence in Adolescents Pediatrics 2007 119 264 274 10.1542/peds.2006-1583 17130279

[B31] Kleinjan M Engels RCME Van Leeuwe J Brug J Van Zundert RMP Van den Eijnden RJJM Examining mechanisms of adolescent smoking cessation: The roles of readiness to quit nicotine dependence and smoking of parents and peers Drug Alcohol Depend 2009 99 204 214 10.1016/j.drugalcdep.2008.08.002 18848408

[B32] Vermulst AA Gerris JRM QBF: Quick Big Five Persoonlijkheidstest Handleiding [Quick Big Five Personality Test Manual] 2005 Leeuwarden the Netherlands: LCD Publications

[B33] Harakeh Z Engels RCME De Vries H Scholte RHJ Correspondence between proxy and self-reports on smoking in a full family study Drug Alcohol Dep 2006 84 40 47 10.1016/j.drugalcdep.2005.11.026 16386380

[B34] Engels RCME Knibbe RA Drop MJ De Haan YT Homogeneity of cigarette smoking within peer groups: Influence or selection? Health Educ Behav 1997 24 799 809 10.1177/109019819702400613 9408792

[B35] Prokhorov AV Pallonen UE Fava JL Ding L Niaura R Measuring nicotine dependence among high risk adolescent smokers Addict Behav 1996 21 117 127 10.1016/0306-4603(96)00048-2 8729713

[B36] DiFranza JR Savageau JA Fletcher K Ockene JK Rigotti NA McNeill AD Coleman M Wood C Measuring the loss of autonomy over nicotine use in adolescents: The DANDY (Development and Assessment of Nicotine Dependence in Youths) study Arch Pediatr Adolesc Med 2002 156 397 403 11929376 10.1001/archpedi.156.4.397

[B37] Kleinjan M Van den Eijnden RJJM Van Leeuwe J Brug J Otten R Engels RCME Factorial and convergent validity of nicotine dependence measures in adolescents: Towards a multidimensional approach Nicotine Tob Res 2007 9 1109 1118 10.1080/14622200701488459 17978984

[B38] Wellman RJ DiFranza JR Savageau JA Godiwala S Savageau N Friedman K Hazelton J Measuring adults' loss of autonomy over nicotine use: The Hooked on Nicotine Checklist Nicotine Tob Res 2005 7 157 161 10.1080/14622200412331328394 15804688

[B39] Kandel D Schaffran C Griesler P Samuolis J Davies M Galanti R On the measurement of nicotine dependence in adolescence: Comparisons of the mFTQ and a DSM-IV-based scale J Pediatr Psychol 2005 30 319 332 10.1093/jpepsy/jsi027 15863429 PMC1282455

[B40] O'Loughlin J Tarasuk J DiFranza JR Paradis G Reliability of selected measures of nicotine dependence among adolescents Ann Epidemiol 2002 12 353 362 10.1016/S1047-2797(01)00312-X 12062924

[B41] Muthen LK Muthen B MPLUS: The comprehensive modeling program for applied researchers 1998 Los Angeles CA: Author

[B42] Cote SM Vailantcourt T LeBlanc JC Nagin DS Tremblay RE The Development of Physical Aggression from Toddlerhood to Pre-Adolescence: A Nation Wide Longitudinal Study of Canadian Children J Abnorm Child Psychol 2006 34 71 85 16565888 10.1007/s10802-005-9001-z

[B43] Otten R Wanner B Vitaro F Van den Eijnden RJJM Engels RCME Disruptiveness peer experiences and adolescent smoking: A long-term longitudinal approach Addiction 2009 104 641 650 10.1111/j.1360-0443.2008.02480.x 19215602

[B44] O'Loughlin J DiFranza J Tarasuk J Meshefedjian G McMillan-Davey E Paradis G Tyndale RF Clarke P Hanley J Assessment of nicotine dependence symptoms in adolescents: a comparison of five indicators Tob Control 2002 11 354 360 10.1136/tc.11.4.354 12432161 PMC1747676

[B45] Karp I O'Loughlin J Hanley J Tyndale RF Paradis G Risk factors for tobacco dependence in adolescent smokers Tob Control 2006 15 3 199 204 10.1136/tc.2005.014118 16728750 PMC2564659

[B46] Wileyto P O'Loughlin J Lagerlund M Meshefedjian G Dugas E Gervais A Distinguishing risk factors for the onset of cravings, withdrawal symptoms and tolerance in novice adolescent smokers Tob Control 2009 18 387 392 10.1136/tc.2009.030189 19648131

[B47] Jaccard J Interaction effects in logistic regression Quantitative application in the social sciences 2001 135 Thousand Oaks CA: Sage Publications

[B48] Byrne DG Byrne AE Reinhart MI Psychosocial correlates of adolescent cigarette smoking: Personality or environment Aust J Psychol 1993 45 87 95 10.1080/00049539308259124

[B49] Carter BL Tiffany ST The cue-availability paradigm: the effects of cigarette availability on cue reactivity in smokers Exp Clin Psychopharmacol 2001 9 183 190 11518094 10.1037//1064-1297.9.2.183

[B50] Brook JS Brook DW Gordon AS Whiteman M Cohen P The psychosocial etiology of adolescent drug use: A family interactional approach Genet Soc Gen Psychol Monogr 1990 116 111 167 2376323

[B51] Saklofske DH Yackulic RA Personality predictors of loneliness Pers Individ Diff 1989 10 467 472 10.1016/0191-8869(89)90011-1

[B52] Conrod PJ Castellanos N Mackie C Personality-targeted interventions delay the growth of adolescent drinking and binge drinking J Child Psychol Psychiatry 2008 49 181 190 18211277 10.1111/j.1469-7610.2007.01826.x

[B53] Conrod PJ Castellanos-Ryan N Strang J Brief, personality-targeted coping skills interventions and survival as a non-drug user over a two-year period during adolescence Arch Gen Psychiatry 2010 67 85 93 10.1001/archgenpsychiatry.2009.173 20048226

[B54] Patrick DL Cheadle A Thompson DC Diehr P Koepsell T Kinne S The validity of self-reported smoking: A review and meta-analysis Am J Public Health 1994 84 1086 1093 10.2105/AJPH.84.7.1086 8017530 PMC1614767

[B55] Stein LA Colby SM O'Leary TA Monti PM Rohsenow DJ Spirito A Riggs S Barnett NP Response distortion in adolescents who smoke: a pilot study J Drug Educ 2002 32 271 286 10.2190/GL7E-B8MV-P9NH-KCVV 12556133 PMC2867081

[B56] Dolcini MM Adler NE Ginsberg D Factors influencing agreement between self-reports and biological measures of smoking among adolescents J Res Adolesc 1996 6 515 542

[B57] Hunter SMD Webber LS Berenson GS Cigarette smoking and tobacco usage behaviour in children and adolescents: Bogalusa study Prev Med 1980 9 701 712 10.1016/0091-7435(80)90015-8 7454695

[B58] Karp I O'Loughlin J Paradis G Hanley J Difranza J Smoking trajectories of adolescent novice smokers in a longitudinal study of tobacco use Ann Epidemiol 2005 15 6 445 452 10.1016/j.annepidem.2004.10.002 15967392

[B59] Koopmans JR Slutske WS Heath AC Neale MC Boomsma DI The genetics of smoking initiation and quantity smoked in Dutch adolescent and young adult twins Behav Genet 1999 29 383 393 10.1023/A:1021618719735 10857244

[B60] Vink JM Willemsen G Boomsma DI Heritability of smoking initiation and nicotine dependence Behav Genet 2005 35 397 406 10.1007/s10519-004-1327-8 15971021

